# PLGA-PVA-PEG Single Emulsion Method as a Candidate for Aminolevulinic Acid (5-ALA) Encapsulation: Laboratory Scaling Up and Stability Evaluation

**DOI:** 10.3390/molecules27186029

**Published:** 2022-09-15

**Authors:** Geisiane Rosa da Silva, Amanda Luizetto dos Santos, Andrey Coatrini Soares, Marinalva Cardoso dos Santos, Sandra Cruz dos Santos, Ştefan Ţălu, Vânia Rodrigues de Lima, Vanderlei Salvador Bagnato, Edgar Aparecido Sanches, Natalia Mayumi Inada

**Affiliations:** 1 São Carlos Institute of Physics (IFSC), University of São Paulo (USP), São Paulo 13560-110, Brazil; 2 Nanomed—Innovation in Nanotechnology, São Paulo 13560-110, Brazil; 3 Embrapa Instrumentation, São Paulo 13560-110, Brazil; 4 Chemical and Food School, Federal University of Rio Grande (FURG), Rio Grande 96203-000, Brazil; 5 The Directorate of Research, Development and Innovation Management (DMCDI), Technical University of Cluj-Napoca, 15 Constantin Daicoviciu St., 400020 Cluj-Napoca, Romania; 6 Laboratory of Nanostructured Polymers (NANOPOL), Federal University of Amazonas (UFAM), Manaus 69067-005, Brazil

**Keywords:** polymeric particles, poly (lactic acid-co-glycolic acid), aminolevulinic acid, polyethylene glycol

## Abstract

One of the most widely used molecules used for photodynamic therapy (PDT) is 5-aminolevulinic acid (5-ALA), a precursor in the synthesis of tetrapyrroles such as chlorophyll and heme. The 5-ALA skin permeation is considerably reduced due to its hydrophilic characteristics, decreasing its local bioavailability and therapeutic effect. For this reason, five different systems containing polymeric particles of poly [D, L–lactic–co–glycolic acid (PLGA)] were developed to encapsulate 5-ALA based on single and double emulsions methodology. All systems were standardized (according to the volume of reagents and mass of pharmaceutical ingredients) and compared in terms of laboratory scaling up, particle formation and stability over time. UV-VIS spectroscopy revealed that particle absorption/adsorption of 5-ALA was dependent on the method of synthesis. Different size distribution was observed by DLS and NTA techniques, revealing that 5-ALA increased the particle size. The contact angle evaluation showed that the system hydrophobicity was dependent on the surfactant and the 5-ALA contribution. The FTIR results indicated that the type of emulsion influenced the particle formation, as well as allowing PEG functionalization and interaction with 5-ALA. According to the ^1^H-NMR results, the 5-ALA reduced the T1 values of polyvinyl alcohol (PVA) and PLGA in the double emulsion systems due to the decrease in molecular packing in the hydrophobic region. The results indicated that the system formed by single emulsion containing the combination PVA–PEG presented greater stability with less influence from 5-ALA. This system is a promising candidate to successfully encapsulate 5-ALA and achieve good performance and specificity for in vitro skin cancer treatment.

## 1. Introduction

Photodynamic therapy (PDT) is a non-invasive technique based on the association of a photosensitizing drug and light application, and the produced reactive oxygen is important for the treatment of oncological diseases [1,2]. Considering the high cost of the non-melanoma skin cancer treatments, the development and improvement of new materials and techniques have been proposed as viable economic alternatives [3,4]. Although topical treatments using PDT associated with hydrophilic prodrugs such as aminolevulinic acid (5-ALA) have already been extensively reported in the scientific literature, some limitations are still considered [5,6,7]. The bioavailability of 5-ALA in skin layers prevents its maximum in loco pharmacological efficiency. For this reason, the nanotechnology-based improvement of this prodrug application and the reduced cost of alternative products have been considered important research topics [5,8].

Polymeric, lipid and metallic particles have been developed for in loco controlled release, improving biodistribution, bioavailability, and therapeutic efficiency and decreasing side effects. In addition, nanotechnology can be associated with different therapies and diagnostic imaging [9]. The development of polymeric nanomaterials presenting high specificity and cost-effectiveness for the development of new pharmaceutical products requires well-defined physicochemical characteristics related to their laboratory scaling up and stability over time [10]. Furthermore, the evaluation of chemical interactions between carriers and encapsulated drugs is equally important, as they can decrease the release efficiency or modify the release mechanism [11,12].

Systems based on polymeric particles of poly (D,L-lactic-co-glycolic acid) (PLGA), although still presenting high commercial value, are clinically approved by the Food and Drug Administration (FDA) [13,14]. PLGA allows topical applications by improving the physicochemical characteristics of several drugs during penetration/retention in skin cells. Furthermore, this polymer is able to protect drugs from interactions with biomolecules, allowing optimization of the treatment processes. A combination with other carriers such as polyethylene glycol (PEG) or deoxyribonucleic acid (DNA) has also been proposed to increase the treatment effectiveness [13,14,15].

The present work proposes the development of five particle systems consisting of PLGA and/or PEG for the encapsulation of 5-ALA based on single and double emulsion methods. Physical properties (organoleptic characteristics, electrical conductivity, relative density and dynamic viscosity) were fully evaluated. Ultraviolet-visible (UV-VIS) spectroscopy, Fourier-transform infrared spectroscopy (FTIR) and proton nuclear magnetic resonance (^1^H-NMR) were performed to investigate the possible chemical interactions between carrier, surfactants and 5-ALA. Nanoparticle tracking analysis (NTA) allowed the obtainment of particle concentration and hydrodynamic diameter. Contact angle and surface energy revealed the influence of surfactants on the systems’ hydrophobicity. Each formulation component was mapped by the ideal mapping technique (IDMAP). Finally, the systems’ stability was evaluated based on the forced stability of 5-ALA and developed systems, as well as the physicochemical stability under different storage conditions.

## 2. Materials and Methods

### 2.1. Materials

All aqueous solutions were prepared using ultrapure deionized water (18.2 M.Ω.cm, Master WFI, Gehaka, São Paulo, Brazil). Acetone, diiodomethane, formamide and ethylene glycol were purchased from LabSynth, Diadema—São Paulo, Brazil. Polyvinyl alcohol (PVA), poly (*D, L*-lactic-co-glycolic acid) [(50:50); Mw = 30,000–60,000 g·mol^−1^] and Kollisolv^®^ PEG400 (polyethylene glycol 400; Mw = 380–420 g·mol^−1^) were purchased from Sigma-Aldrich (Burlington, MA, USA). Aminolevulinic acid hydrochloride [95% and 99% (5-ALA)] were purchased from PDTPharma (Cravinhos, São Paulo, Brazil).

### 2.2. Particle Development

The developed systems were standardized to a final volume of 190 mL and 0.4478 mg·mL^−1^ (29.38% and 53.13% of 5-ALA in relation to the mass of PLGA for single and double emulsion systems, respectively). The final volumes were 260 mL and 308 mL for double (Systems A and E) and single (Systems B, C and D) emulsions, respectively.

The mass ratio was determined according to the patents N° US6,559,183 (AmeLuz^®^, Biofrontera Inc., Woburn, MA, USA) [16] and N° WO2011156880A1 [17]. System preparations are described as follows:

**System A:** (1) The organic phase was prepared using PLGA [(50:50); (0.16000 ± 0.00001) g] solubilized in acetone (2.318 mg·mL^−1^) at 25°C for 15 min under magnetic stirring at 750 rpm (IKA C–MAG +HS7). (2) The internal phase was prepared using deionized water (82 mL) and 5-ALA (1.0366 mg·mL^−1^; 53.13% *w*/*w* in relation to the mass of PLGA) for 15 min under magnetic stirring at 750 rpm. (3) The internal phase was added into the organic phase (pre-emulsification) in an ice bath under magnetic stirring at 3200 rpm (Ultra Turrax^®^ T–25 digital) for 20 min. (4) The external phase was prepared using deionized water (109 mL) and PVA (1% *w*/*v*) under magnetic stirring at 45 °C for 50 min. Then, the PVA solution was submitted to ice bath (4 °C) under magnetic stirring at 750 rpm. (5) The emulsion obtained in (3) was added to the external aqueous phase under magnetic stirring at 1500 rpm for 30 min. The final concentration of 5-ALA was (0.4474 ± 0.0002) mg·mL^−1^. The active concentration was standardized for all syntheses in relation to the final volume of particles. The synthesis without 5-ALA (unloaded systems) was prepared under the same conditions. Solvent evaporation was obtained on a rotary evaporator (Heidolph, Hei-VAP Advantage) at 120 rpm, 35 °C and 40 mbar.

**System B:** (1) The organic phase was obtained using PLGA (50:50); (0.28500 ± 0.00001) g solubilized in acetone (2.4 mg·mL^−1^) at 25°C for 15 min under magnetic stirring at 750 rpm. (2) The Kolliphor solution (PEG) [1% (*w*/*v*)] was obtained under magnetic stirring at 750 rpm and cooled in ice bath (4 °C). Then, 5-ALA was solubilized in the aqueous phase with PEG under magnetic stirring at 750 rpm for 15 min (maintaining 29.36% of 5-ALA in relation to the mass of PLGA). (3) The aqueous phase was added to the organic phase under magnetic stirring at 1500 rpm for 30 min in ice bath (0–4°C). (4) Solvent was removed.

**System C:** (1) The organic phase was obtained using PLGA (50:50) solubilized in acetone at room temperature under stirring at 750 rpm for 15 min (under the same conditions described in Synthesis B). (2) The aqueous phase was prepared using PVA [0.5% (m/v)] under magnetic stirring at 45 °C for 50 min. Then, the PVA solution was cooled in ice bath (4 °C) under magnetic stirring at 750 rpm. PEG [0.5% (m/v)] was added under the same magnetic stirring and temperature for 15 min. The 5-ALA was added in the aqueous phase (under the same described conditions), maintaining 29.36% of 5-ALA in relation to the mass of the PLGA. (3) The aqueous phase was added to the organic phase under magnetic stirring at 1500 rpm for 30 min in ice bath (0–4 °C). (4) Solvent was removed.

**System D:** (1) The organic phase was obtained using PLGA (50:50) solubilized in acetone at room temperature under magnetic stirring at 750 rpm for 15 min (under the same conditions described in Synthesis B). (2) The external phase was prepared with deionized water (190 mL) and PVA [1% (m/v)] under magnetic stirring at 45 °C for 50 min. Then, the PVA solution was cooled in ice bath (4 °C) under magnetic stirring at 750 rpm. Then, 5-ALA was added in the proportion of 29.36% in relation to the mass of PLGA (under the same magnetic stirring conditions and temperature) for 15 min. (3) The aqueous phase was added into the organic phase under magnetic stirring at 1500 rpm for 30 min in ice bath (4 °C). (4) Solvent was removed.

**System E:** (1) The organic phase was obtained using PLGA (50:50) solubilized in acetone (under the same conditions described in Synthesis A). (2) The internal phase was formed by PVA [0.5% (m/v); 82 mL] under magnetic stirring at 750 rpm at 55 °C and cooled in ice bath (0–4 °C). Then, 5-ALA was solubilized in the internal phase (under the same conditions described in Synthesis A) under magnetic stirring at 750 rpm for 15 min. (3) The internal phase was added to the organic phase (pre-emulsification) in ice bath under magnetic stirring at 3200 rpm for 20 min. (4) The external aqueous phase was obtained using PEG [0.5% (m/v)] under magnetic stirring at 750 rpm at room temperature for 15 min. (5) Then, the pre-emulsion was added to the external aqueous phase in ice bath under magnetic stirring at 1500 rpm for 30 min, allowing the nanoemulsification. (6) Solvent was removed.

### 2.3. System Characterization

#### 2.3.1. Physical Properties

Electrical conductivity was determined using a conductivity meter (Sensoglass, São Paulo, Brazil) ranging from 0.001 mS to 500 mS and sample volume of 15 mL. The relative density was determined indirectly using an analytical balance and a 1000 µL micropipette measuring the relative mass of 1 mL of each system. Viscosity was determined using a microprocessor rotary viscometer (Q860M26, Quimis, Diadema—São Paulo, Brazil) using 15 mL of the systems considering viscosity similar to that of water. Measurements were performed in triplicate.

#### 2.3.2. Ultraviolet-Visible (UV-VIS) Spectroscopy

Absorbance measurements were performed on a U–2900 UV-VIS spectrophotometer (Hitachi, Tokyo, Japan) using a quartz cuvette (optical path of 1 cm) from 200 to 1100 cm^−1^.

#### 2.3.3. Nanoparticle Tracking Analysis (NTA)

Size characterization was performed on a NanoSight NS300 device (Malvern Instruments, Malvern, UK). Data collection and analysis were performed using the software NTA 3.0. Samples were diluted in MilliQ water (1:10,000 *v*/*v*). Measurements were performed in triplicate at 25 °C. The evaluation of the particle size distribution (PSD) was performed through the parameters of average size, mode and standard deviation.

#### 2.3.4. Dynamic Light Scattering (DLS) and Zeta Potential

Size and polydispersity evaluations were also performed on Zetasizer Nano ZS90 equipment (Malvern Instruments, Malvern, UK). Unloaded and loaded systems were diluted in deionized water [(1:10), (1:100) and (1:1000) *v*/*v*]. Size distribution and polydispersity index (PDI) measurements were obtained at 25 °C and a scattering angle of 173°. Measurements were performed in quintuplicate. Surface charges of the unloaded and loaded systems were measured at 25 °C using a DTS1070 capillary cell and the same dilution described previously. Measurements were performed in quintuplicate.

#### 2.3.5. Contact Angle and Surface Energy

Surface tension (*γ*) parameters for hydrophobicity and surface energy calculations are shown in Table 1 [18]. Polar and dispersive components were determined from the contact angle and surface energy using the sessile drop method. Ten glass slides were prepared with five drops ((0.8 ± 0.1) µL for each drop) of each solution and allowed to dry in a controlled atmosphere. Prob liquid drops ((3.0 ± 0.1) µL, Table 1) were deposited on the surface of the dried drops. Angles were calculated from the average of five drops photographed at 10 frames.s^−1^ using the software CAM 200. Measurements were performed on a KSV model CAM–200 goniometer (KSV Instruments).

#### 2.3.6. Fourier-Transform Infrared Spectroscopy (FTIR)

FTIR spectra were obtained on a Shimadzu-IR Prestige–21 (Shimadzu, Kyoto, Japan) spectrophotometer in horizontal attenuated total reflection (HATR) mode. Samples (1 mL) were placed on a zinc selenide crystal, and interferograms were from 4000 cm^−1^ to 400 cm^−1^ using 45 scans and resolution of 2 cm^−1^.

#### 2.3.7. Proton Nuclear Magnetic Resonance (^1^H-NMR)

The ^1^H-NMR data were obtained at 23 °C on a Bruker Avance 400 instrument (Bruker, Billerica, MA, USA) using 400 MHz. Trimethylsilylpropanoic acid was used as reference, and samples were diluted in deuterated water (80:20). The pre-saturation pulses were applied to the suppression of the water peak. The inversion–recovery pulse sequence (180°–90°) was used to obtain the relaxation time (T1) values of specific methylene peaks. The acquisition time and relaxation delay were, respectively, 4 s and 10 s. A 32 transients average was obtained for each time delay, and 16 values were distributed from 0.001 s to 10 s. The T1 values and relative intensities were calculated by fitting the exponential data using the software NUTS.

### 2.4. System Stability

Stability evaluation was based on the ANVISA National Health Surveillance Agency) [19] and FDA (U.S. Food and Drug Administration, Silver Spring, Maryland) [20] regulations. Changes of up to 15% were considered stable when compared to the initial evaluated parameters. The reproducibility of the physicochemical measurements was based on the RE N°1 standard (07/29/2005) from ANVISA and Resolution of the Collegiate Board (RDC) N° 318 (11/06/2019), which establishes the criteria for carrying out stability studies of active pharmaceutical ingredients and medicines, as follows.

#### 2.4.1. Forced Stability of 5–ALA

A solution of ultrapure water (50 mL; 0.4472 mg.mL^−1^) and 5-ALA was prepared. This solution was divided into pharmaceutical glass vials and stored at (23.0 ± 2.0) °C, (5.0 ± 1.0) °C, (40.0 ± 2.0) °C and cycled (24 h stored at (5.0 ± 1.0) °C and 24 h at (40.0 ± 2.0) °C). The pH measurements were performed for four consecutive days in triplicate.

#### 2.4.2. Forced Stability of Nanoemulsions (3000 rpm at 56 °C)

The systems were centrifuged (Ultra Centrifuge Eppendorf 5427, Barkhausenweg, Hamburg, Germany) at 24 °C using three cycles of 30 min. The nanoemulsions were evaluated at the end of each cycle according to their organoleptic properties and pH variation. Samples were discarded after analysis. Measurements were performed in triplicate.

An aliquot of 15 mL was placed in 100 mL regent flasks, which were closed and covered with aluminum foil. All vials were maintained in an oven for 48 h at (56.0 ± 2.0) °C. Stability was evaluated according to organoleptic properties, zeta potential, particle size and pH parameters. Samples were discarded after analysis. All measurements were performed in quintuplicate.

#### 2.4.3. Physicochemical Stability under Storage Conditions

A volume of 30 mL of each system was stored in a transparent borosilicate vial with head space for possible gas exchange. The vials containing the loaded systems were covered with aluminum foil.

Systems were stored at (23.0 ± 2.0) °C, (5.0 ± 1.0) °C, (40.0 ± 2.0) °C and cycled (24 h stored at (5.0 ± 2.0) 1.0) °C and 24 h at (40.0 ± 2.0) °C). All storage conditions were analyzed for 40 days according to their pH values, and after 1, 7, 15, 30 and 40 days according to particle size, PDI and surface charge. The cycled samples were discarded. However, the systems’ storage under the first three conditions was evaluated after 60, 90, 120, 150 and 180 days (preliminary stability), as well as after 360, 540 and 720 days (shelf-life test). Systems presenting non-reproduced organoleptic and physicochemical properties were discarded.

### 2.5. Data Analysis

Statistical analysis was performed using the Projection Explorer Sensors (PEx-Sensors), a Java-based tool used to create visual representations of data collected by sensors [21]. Data from particle size, zeta potential, PDI and pH were projected on a 2D map to evaluate the similarity of the systems according to their physicochemical changes. The interactive document map (IDMAP) technique uses Euclidean distance *δ*(*x_i_, x_j_*) between the signals of different samples *X* = {*x*_1_*, x*_2_*, …, x_n_*} to project data into a smaller dimensional space. In this space, the positioning of visual elements *Y = {y*_1_*, y*_2_*, …, y_n_}* is given as *f*: X → Y, which minimizes the term |*δ*(*x_i_*,*x_j_*) – *d*(*f*(*x_i_*), *f*(,*x_j_*))|*∀ x_i_*,*x_j_* ∈ *X*. Function is given by the following equation [22]:

(1) $$S_{IDMAP}=\frac{\delta \left(x_{i},x_{j}\right)-\delta_{min}}{\delta_{max}-\delta_{min}}-d\left(y_{i},y_{j}\right)$$

where *δ_max_* and *δ_max_* are, respectively, the maximum and minimum Euclidean distances between data instances, and *d*(*y_i_, y_j_*) represents the Euclidean distance between the instances projected in the smallest dimensional space.

## 3. Results and Discussion

### 3.1. Particle Development

System A (Table 2) consisting of PLGA particles was reported previously based on the double emulsion method [23]. According to the authors, the active particles presented average size of 249.5 nm and were obtained by solubilizing PLGA in dichloromethane, as well as an internal aqueous phase formed by PBS buffer. The reported results of the in vitro and in vivo tests are promising for the application in PDT, and these results were our initial criteria for the choice of this system. For this reason, we proposed here a simple emulsion method for this system, which was labelled as Synthesis D (Table 2). The objective of developing this system was to compare it with that of System A, aiming to enhance its stability, as well as simplifying the development method. The systems analyzed under the following stability tests are described in Table 2.

We aimed to favor in vivo and in vitro applications from the use of PVA as a surfactant. For this reason, particles functionalized or covered with low molecular weight poly(ethylene glycol) (PEG) [24] were developed: System C and System E (Table 2) were developed based on the single and double emulsion methods, respectively.

Finally, System B (Table 2) contained only PEG as a surfactant. It is known that PEG is not an efficient active carrier. Therefore, this system was developed for comparison to the Systems C and E in order to verify the influence of each component on the formation and stability of the developed particles [25]. Thus, we propose here some adjustments in system development, aiming for innovation in the methodology of obtaining PLGA particles for the encapsulation of 5-ALA.

#### Laboratory Scaling Up

The use of PLGA is approved by the Food and Drug Administration (FDA) and the European Medicines Agency (EMA) as a polymer carrier for controlled release of molecules with therapeutic potential. However, few regulatory studies related to their stability in large-scale production [26,27]. For this reason, we evaluated here the laboratory scaling up of the developed systems by increasing volume and mass (more than six times) of the reagents in relation to the initial parameters.

**Figure 1:**
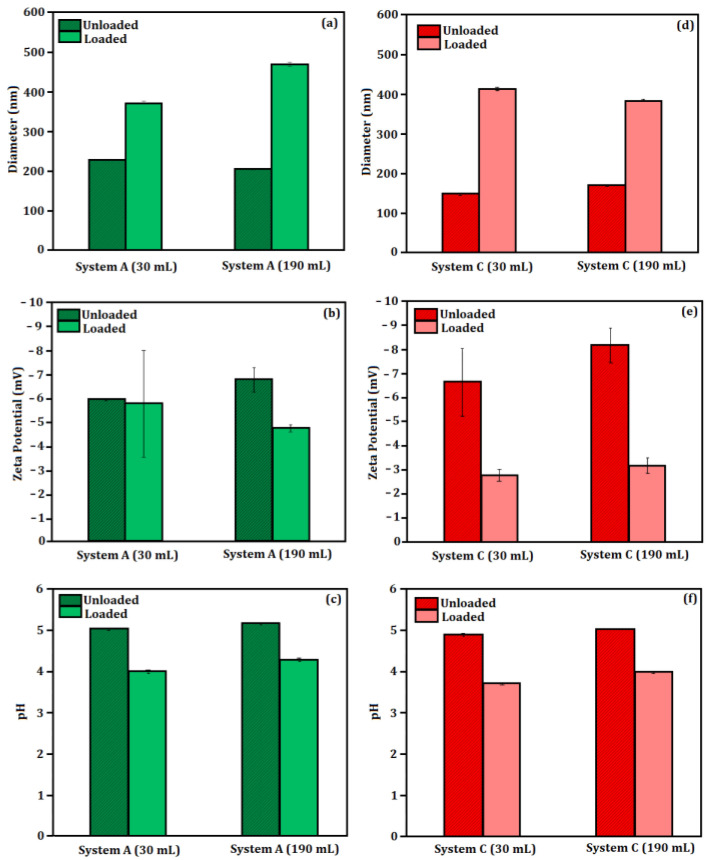
Laboratory scaling up from 30 mL to 190 mL of Systems A and C considering (**a**) diameter of System A, (**b**) diameter of System C, (**c**) zeta potential of System A, (**d**) zeta potential of System C, (**e**) pH of System A and (**f**) pH of System C.

Figure 1 shows the evaluation of particle size (nm), zeta potential (mV) and pH of Systems A and C. Considering System A, Figure 1a shows a loaded particle diameter increasing around 25.96%, while the diameter of the unloaded particles decreased by 10.37%. Figure 1b shows the increase in zeta potential in the unloaded particles, and Figure 1c shows pH variations between 2.57% and 6.96%, respectively, in loaded and unloaded systems. Considering System C, Figure 1d shows an increase in diameter of around 14.45% in unloaded particles, while the loaded systems presented a reduction of 7.17% in diameter. Figure 1e shows the increase (in module) in zeta potential by around 22.71% and 14.80%, respectively, in unloaded and loaded nanoparticles. Finally, Figure 1f shows that the basicity was increased by 2.45% and 7.56%, respectively, in the unloaded and loaded particles.

The particle diameters of Systems A and C presented variations as a function of volume solution and addition of 5-ALA. In general, unloaded particles were smaller than those containing encapsulated bioactives. This behavior was maintained in the scaled systems, and the increase in particle diameters may be related to the 5-ALA concentration [28].

The concentrations of PLGA (50:50) in System A considering 30 mL and 190 mL were (0.01469 g.mL^−1^) and (4.13 × 10^−3^ g.mL^−1^), respectively. This fact could explain the decrease in the unloaded particle diameter of the scaled-up system. The same result was observed in System C; however, the polymer concentration in the loaded particles was maintained at 2.4 × 10^−3^ g.mL^−1^ in both 30 mL and 190 mL solutions [29].

The calculation of the surfactant concentration was different for the simple and double emulsion systems. The percentage of PVA (1%) in the double emulsion system was calculated for one of the aqueous phases, while the concentrations of 0.5% (PVA) and 0.5% (PEG) were calculated for the total volume of the aqueous phase. For this reason, in the scaling up process, an increase in the polymer concentration gradient (from the organic phase to the aqueous phase) was expected as a function of the increased water volume. Then, a particle size decrease was observed in the loaded particles from the simple emulsion system [29].

Considering the zeta potential evaluation, there was a tendency toward decreasing values after the addition of 5-ALA. This result was probably related to the dependency between surface charge and the bioactive, and it was also observed in the pH values of the loaded and unloaded systems: the values were increased as a function of the solution volumes [28].

Nanoemulsions prepared on a laboratory scaling up are usually difficult to be converted to industrial production mainly due to the differences in the laboratory instruments [27]. The type of homogenization is also significant to obtain monodisperse systems, in addition to the choice of the formulation ingredients. However, it is important to highlight that the homogenization methods are simple and can facilitate a scaling up larger than 2500 mL of emulsion [30].

Our results showed the feasibility of the laboratory scaling up process, considering the performed adaptations. For this reason, all developed systems were standardized (as a function of the volume of reagents and mass of formulation ingredients) to be compared in terms of formation, physicochemical properties and stability.

### 3.2. Characterization

#### 3.2.1. Organoleptic Properties, Electrical Conductivity, Relative Density and Dynamic Viscosity

A precipitate was observed after the nanoemulsion obtainment, and a smaller amount of this residue was observed in System C. This residue was removed after the acetone rotaevaporation process, which may have resulted in some mass loss of 5-ALA during solubilization. After the rotaevaporation process, a translucent characteristic was observed in the unloaded particle systems. The loaded systems presented an opaque appearance. The loaded and unloaded particles from System B presented homogeneous and fully transparent phases. None of the systems presented odor or phase separation up to 90 days. However, Systems A and D presented a white precipitate after 120 days and, after homogenization, these systems returned to a single phase. After 160 days, System C also presented a precipitate and also remained uniform after homogenization.

**Figure 2:**
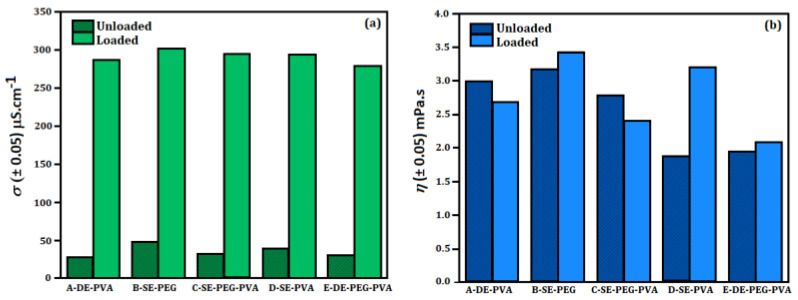
(**a**) Electrical conductivity (*σ*) and (**b**) dynamic viscosity (*η*) of System A (A–DE–PVA), System B (B–SE–PEG), System C (C–SE–PEG–PVA), System D (D–SE–PVA) and System E (E–DE–PEG–PVA).

Figure 2 shows the electrical conductivity (*σ*) and dynamic viscosity (*η*) of the developed systems. The addition of 5-ALA influenced the physical properties, increasing the electrical conductivity and viscosity.

The relative density of all systems was found to be around (0.9982 g·mL^−1^) at 20 °C, and the addition of 5-ALA did not influence the density values. PLGA particles loaded with cisplatin showed increased density after encapsulation [31]. Furthermore, PLGA (50:50) powder nanoparticles (Degradex^®^ PLGA; 30,000 g·mol^−1^; average size of 100 nm; Sigma-Aldrich) are commercialized with relative density of 1.3 g·mL^−1^. For this reason, the relative density is dependent on the formulation ingredients.

According to Figure 2a, the electrical conductivity was higher in all systems containing loaded particles. In addition, System B presented higher electrical conductivity for both loaded and unloaded particles. An increased difference of 42.88% in electrical conductivity was observed in the unloaded Systems A and B. PVA films were reported presenting electrical conductivity of 2.78 × 10^−3^ μS·cm^−1^ [32], while PLGA particles loaded with cisplatin showed electrical conductivity between 3.65 μS·cm^−1^ and 13.52 μS·cm^−1^ [31] as a function of the mass of PLGA and active for unloaded and loaded particles, respectively. The reported differences may be related to the components of the formulation and to the types of particles. The increase in electrical conductivity of the loaded systems was expected. Hydrochlorides are common in several types of drugs, including 5-ALA. Generally, drugs originate from weak bases/acids and are obtained as salts to increase hydroficity (as well as dissolution and absorption in the target of application), increasing their therapeutic efficiency. For this reason, the ionization of 5-ALA takes place in water (amine base), HCl → H^+^ + Cl^−^ [33].

PVA is not a good electrical conductor due to the possible physical interactions with hydroxyl groups, in addition to favoring the formation of complexes. However, its electrical conductivity is improved by associating it with other polymers such as PEG [34]. This association results in miscible materials, with hydrogen bonds occurring between the hydroxyl groups of PVA and –C–O–C– of PEG [35]. This characteristic may contribute to an increase in the electrical conductivity of the human skin (which is a measure of permeability) during drug delivery. The increase in the ion mobility in human skin may improve the transdermal delivery of 5-ALA proportionally to the increase in the drug molecular flux [36]. Reports showed that PEG increased the flux of clonazepam and lorazepam through excised skin. In addition, the combination of anionic and cationic surfactants increased the skin conductivity and drug permeability [37]. However, the evaluation of other parameters such as viscosity, pH and surface charge is required.

System viscosity may change according to the formulation ingredients, polymer concentration and type of active. As shown in Figure 2, the dynamic viscosity values were higher than that of water (1.03 ± 0.05 mPa·s, theoretical value of 1.005 mPa·s). System B (B–SE–PEG), System D (D–SE–PVA) and System E (E–DE–PEG–PVA) presented increased viscosity when 5-ALA was encapsulated. System B (B–SE–PEG) and System D (D–SE–PVA) showed higher viscosity. The presence of chlorhydrate also influenced the viscosity due to the increase in ions. Consequently, an increased particle size was observed [38]. PLGA particles loaded with cisplatin presented viscosity from 1.62 mPa·s to 1.87 mPa·s as a function of PLGA and active mass [31], directly influencing the formation of particles.

#### 3.2.2. UV-VIS Spectroscopy

**Figure 3:**
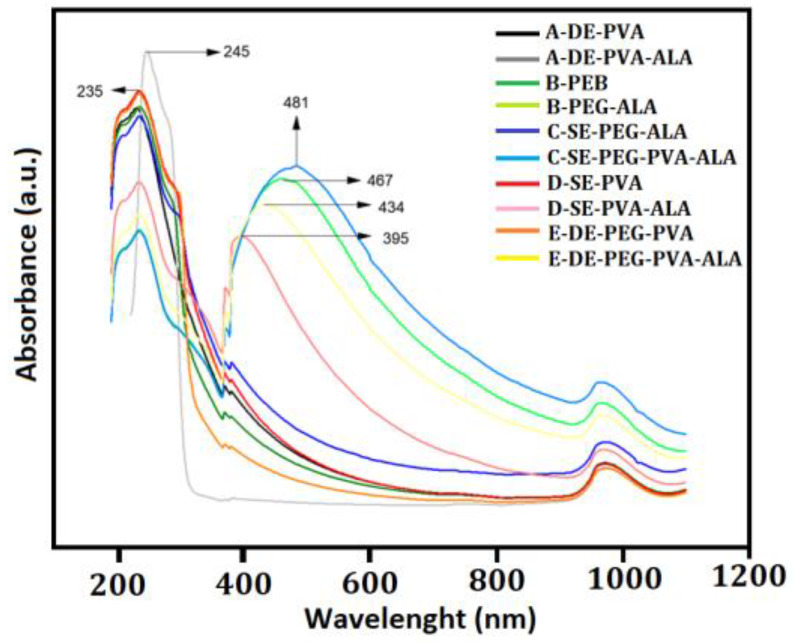
UV-VIS spectroscopy of the unloaded and loaded systems.

According to Figure 3, the contribution of the band around 400 nm in Systems B, C, D and E was clear, suggesting interactions with 5-ALA. This interaction was not observed in System A probably due to (i) the low mass of 5-ALA interacting with the particles (as observed at 245 nm), showing no significant structure changes, or (ii) the ability of 5-ALA to be loaded within the particles or remain unloaded. In addition, PEG could interact more efficiently in the 400 nm region since the encapsulated system showed a peak shift to 395 nm. The pure PVA did not show absorption in the region of 200–800 nm; however, it showed absorbance around 200 nm in aqueous solution. The peak at 194 nm was assigned to the –C=O and –C=C groups; additionally, a displacement to 197 nm was associated with residual acetate groups (negative ion) [39,40,41]. PEG exhibited the O–C–H bond contributing to the bands around 200–400 nm [42]. The contribution of the –C=O bond from PLGA (50:50) was found around 220–270 nm, and the greater wavelength may be related to the particles [43,44]. The absorption around 400–500 nm suggested an interaction between PLGA (50:50) and 5-ALA [45]. Therefore, the UV-VIS spectra showed that the systems’ absorption was dependent on their method of synthesis and may result in the absorption or adsorption of 5-ALA.

#### 3.2.3. Nanoparticle Tracking Analysis (NTA)

The NTA evaluation allows the evaluation of the concentration and hydrodynamic diameter of particles in suspension, which can be compared to DLS results. Table 3 shows the mode and average hydrodynamic diameter obtained by NTA and compared to that obtained by DLS. 

Differences in size distributions between DLS and NTA techniques were observed in System A (A–DE–PVA–ALA), System D (D–SE–PVA–ALA) and System E (E–DE–PEG–PVA–ALA). The NTA and DLS analyses are based on the Brownian movement of particles. However, the NTA technique accounts for the diffusion coefficient, so each particle size is determined, allowing greater size distribution accuracy. On the other hand, the DLS technique analyzes the time-dependent scattered light intensity signal from a single detector. For this reason, it does not have enough resolution to differentiate size populations, resulting in a single Gaussian distribution formed by particles with small differences in size.

The dilution difference (10^3^ and 10^2^, respectively, for NTA and DLS) is an important parameter. However, the distribution curve of System B (B-SE-PEG and B-SE-PEG-ALA) was not obtained due to the low particle concentration [46,47]. Moreover, 5-ALA increased the particle size as well as the functionalization and adsorption of PEG. System C presented the highest particle concentration (in the order of 10^13^), resulting in an increase in absorption around 480 nm, as observed by UV-VIS spectroscopy. This result is important in cell culture analysis because the initial particle concentration does not influence the highest tested concentrations [48]. The standard deviation from the NTA results was smaller than that obtained from DLS analysis probably due to the number of collected statistical data. However, both techniques are complementary [46,47].

#### 3.2.4. Contact Angle and Surface Energy

**Figure 4:**
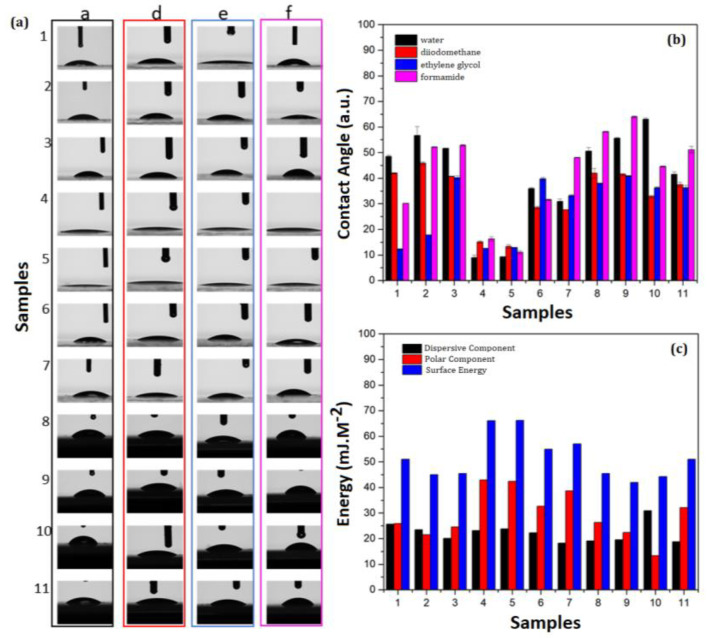
(**a**) Representation of the contact angle, (**b**) variation of the contact angle for the liquid-probes water, diiodomethane, ethylene glycol and formamide, and (**c**) surface energy and its polar and dispersive components for (1) glass, (2) A–DE–PVA, (3) A–DE–PVA–ALA, (4) B–SE–PEG, (5) B–SE–PEG–ALA, (6) C–SE–PEG–PVA, (7) C–SE–PEG–PVA–ALA, (8) D–SE–PVA (9)D–SE–PVA–ALA, (10) E–DE–PEG–PVA and (11) E–DE–PEG–PVA–ALA.

The contact angle visualization and the contact angle variation of each liquid-probe are shown, respectively, in Figure 4a,b.

Hydrophilicity was clear in System B (Figure 4b), reducing the contact angle about 80% in relation to water. The presence of 5-ALA decreased the contact angle in most systems. However, Systems B and D presented contact angles similar to those of unloaded systems. In addition, the presence of 5-ALA implied a change of 34% to System E. Thus, Systems A and D containing 5-ALA presented similar hydrophilicity, and System C presented a reduced contact angle of approximately 36% in relation to water.

For diiodomethane, only System E containing 5-ALA showed an increased contact angle about 14%. In the other systems, 5-ALA decreased the contact angle in relation to the unloaded particles. Systems A and D containing 5-ALA showed hydrophilicity similar to that of the used solvent. For this reason, System B was the most hydrophilic, followed by System C containing 5-ALA (which presented a reduced contact angle by 34% in relation to the unloaded system).

For ethylene glycol, System B presented polarity similar to that of the liquid-probe, and the 5-ALA marginally influenced this interaction. Regarding the unloaded particles, Systems A and D presented similarly increased contact angles. However, the contact angles decreased in Systems C and E, besides showing a lower influence from 5-ALA on System E.

For formamide, the contact angle increased in all loaded particles, except in System B. The 5-ALA presented a marginal influence on System A, and the greatest influence was observed in System C (~16%). System D was the most hydrophobic loaded system, presenting an increased contact angle (~47%). Despite considering PLGA as a hydrophobic polymer, as all contact angles were less than 90° (and less than 65° specifically for water), all PLGA particle surfaces were hydrophilic.

The contact angle results showed that the hydrophobicity of the systems was dependent on the surfactant. The contribution of 5-ALA was observed because the change in the surface chemistry significantly influenced the wettability (hydrophilic or hydrophobic). The –OH polar functional groups interacted more easily with hydrophilic polymers (PVA and PEG), increasing the wettability of particle films and decreasing their surface tension [49].

The 5-ALA presented less influence on Systems B and D, and Systems C and E presented similar dispersivity. However, Systems A and D presented lower polarity. The presence of 5-ALA did not influence System B polarity. Therefore, the surface energy increased as a function of polarity (hydrophobicity), as observed in the systems containing PEG.

The particle surfaces containing only PVA presented lower polarity and surface energy. Considering the PEG–PVA combination, the surface energy was significantly related to the presence of PEG. However, the influence of 5-ALA on the surface energy was clear because PEG can increase the repulsive steric interactions and prevent particle destabilization, in addition to functionalization. A previous report showed that the stabilization of pluronic F–127 (non-ionic surfactant) also modified the surface energy of PLGA particles [50].

The surface tension of human skin is found to be around 27–28 mJ·m^−2^. For this reason, transdermal formulations presenting similar values can facilitate particle adherence/permeation [51]. Despite presenting higher surface energy values, the association of surfactants as well as the incorporation of nanoparticle solutions into pharmaceutical bases must be considered. A previous report showed the interaction of PLGA particles at fluid interfaces, as well as the influence of non-ionic surfactants such as pluronic F–127 on the penetration of particles into lipids [50]. For this reason, the PVA–PEG combination is expected to enhance skin permeation.

#### 3.2.5. Fourier-Transform Infrared Spectroscopy (FTIR)

**Figure 5:**
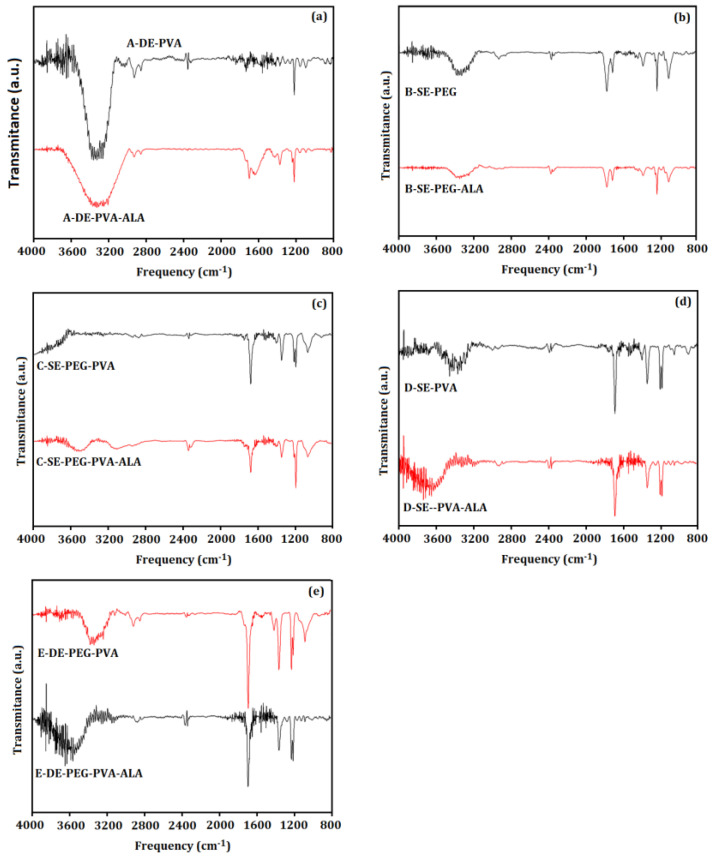
FTIR spectra of Systems (**a**) A–DE–PVA and A–DE–PVA–ALA, (**b**) B–SE–PEG and B–SE–PEG–ALA, (**c**) C–SE–PEG–PVA and C–SE–PEG–PVA–ALA, (**d**) D–SE–PVA and D–SE–PVA–ALA, (**e**) E–DE–PEG–PVA and E–DE–PEG–PVA–ALA.

Figure 5 shows the FTIR spectra of System A (A–DE–PVA and A–DE–PVA–ALA), System B (B–SE–PEG and B–SE–PEG–ALA), System C (C–SE–PEG–PVA and C–SE–PEG–PVA–ALA), System D (D–SE–PVA and D–SE–PVA–ALA) and System E (E–DE–PEG–PVA and E–DE–PEG–PVA–ALA). 

Figure 5a shows a peak at 3321 cm^−1^ assigned to the hydroxyl group of polyvinyl alcohol. The hydroxyl peak of System A (A–DE–PVA) disappeared in the presence of 5-ALA in the A–DE–PVA–ALA system, indicating a possible chemical interaction [52]. Furthermore, PVA presented typical bands of *ν_as_*C–O and *ν_s_*C–O, respectively, at 1219 cm^−1^ and 1097 cm^−1^ [52]. The peak related to *ν_s_*C=O was found at 1697 cm^−1^ in the spectrum of the unloaded double emulsion system.

The addition of 5-ALA in the double emulsion system influenced the frequency and bandwidth of *ν*C–O and *ν_s_*C=O, showing that the active affected the ester region of the double emulsion systems. The active-induced variation frequency of *ν_s_*CH from 2924 cm^−1^ to 2926 cm^−1^ indicated an increase in the gauche bond in the methylene region [53] due to the decrease in the van der Waals interactions. For this reason, the distance between methylene chains was increased, and the molecular packing was decreased [53,54].

Figure 5d shows a broad peak at 3300 cm^−1^ assigned to the –OH group due to the typical intra– and intermolecular hydrogen bonding [55,56], which occurs between PVA chains due to their higher hydrophilic characteristic [56]. Furthermore, the band at 1222 cm^−1^ was related to the *n_as_*O–C–C vibration [52,56]. Moreover, the presence of 5-ALA increased the intensity of the –C=O, –OH and –O–C–C bands, as well as the *ν_s_*CH_2_ and *ν_as_*CH_2_ [57]. The decreased frequency of the *ν_s_*CH_2_ and *ν_as_*CH_2_ bands, as well as the reduction in their bandwidth, indicated lower isomerization and methylene movement induced by 5-ALA. These results suggested a parallel organization of the methylene groups stabilized by the van der Walls interactions due to the presence of 5-ALA [54,58]. Furthermore, the decreased frequency of the *ν*C=O band showed that 5-ALA did not influence on the degree of hydration related to the number of hydrogen bonds [59,60,61]. However, the 5-ALA-induced decrease in the *ν*C=O bandwidth suggested a restriction of the carbonyl molecular movement. Finally, the influence of 5-ALA on the ester region was also suggested due to the decrease in the *ν*C–O–C bandwidth. Therefore, the spectra of Systems A and D revealed that 5-ALA influenced the systems’ connections. Furthermore, a significant interaction of the active was observed in the simple emulsion system. The 5-ALA was adsorbed on the particle surfaces, and the type of emulsion may have favored different connections where PVA significantly interacted in the systems presenting bonds formed with the active.

Figure 5b shows that the interaction of polymers with 5-ALA increased the *ν_s_*CH_2_ and *ν_as_*CH_2_ frequency, indicating increased *trans*-gauche isomerization due to the core shell interactions. The increased *ν_as_*CH_2_ bandwidth suggested a lower restriction of the movement of methylenes because this band is more sensitive to mobility than *ν_s_*CH_2_. On the other hand, the increased *ν*C=O bandwidth suggested higher mobility of the carbonyl group induced by the 5-ALA. No significant active interaction was related to the *ν*C–O–C group, as expected. The band of the PVA–OH group from System C–SE–PEG–PVA was not observed (Figure 5c), indicating a strong interaction with PEG. These results suggested that PEG allowed the particle functionalization. The overlapped methylene bands (*ν_s_*CH_2_ and *ν_as_*CH_2_) were observed at 2949 cm^−1^ in the spectrum of System C–SE–PEG–PVA–ALA. Before interaction with 5-ALA, the band elongation of the hydroxyl group was verified at 3518 cm^−1^. After interaction, this band shifted to 3115 cm^−1^. No changes were observed in the *n*C=O band after the encapsulation of 5-ALA, but a discrete interaction of the active with the –C–O–C group was observed due to the decreased *n*C–O–C bandwidth.

The spectrum of System E–DE–PEG–PVA showed the –OH band at 3325 cm^−1^ in both PVA and PEG molecular structures (Figure 5e). The discrete shoulder at 1093 cm^−1^ was assigned to the *ν*C–O band of PVA and PEG. The variation in the *ν*C–O bandwidth indicated the influence of 5-ALA on the interfacial region of the double emulsion. The increased *ν_as_*CH bandwidth showed that 5-ALA increased the mobility of the hydrophobic region, as observed previously in the contact angle and surface energy results [60]. In addition, no hydroxyl group was found attached to 5-ALA.

The carrier PEG influenced the mobility of the –CH band in System E–DE–PEG–PVA (Figure 5e), suggested by the reduced *ν_as_*CH bandwidth. The ester region was also affected because a reduced *ν*C-O bandwidth was observed [60]. A typical hydroxyl group band was observed in the spectra of Systems E–DE–PEG–PVA (Figure 5e) and D–DE–PVA (Figure 5a), indicating that PEG did not influence the bonds between the hydroxyl groups of PVA and an external agent [52]. Furthermore, the insertion of PEG in System E–DE–PEG–PVA affected only the interface, suggested by the reduced bandwidth of the *ν*C–O band. For this reason, the hydroxyl group was not linked to another agent due to the unchanged shoulder of the hydroxyl band. Our results indicated that the type of emulsion influenced the particle formation, as well as allowing the PEG functionalization and interaction of the 5-ALA on their surfaces.

#### 3.2.6. H-RMN Analysis

**Figure 6:**
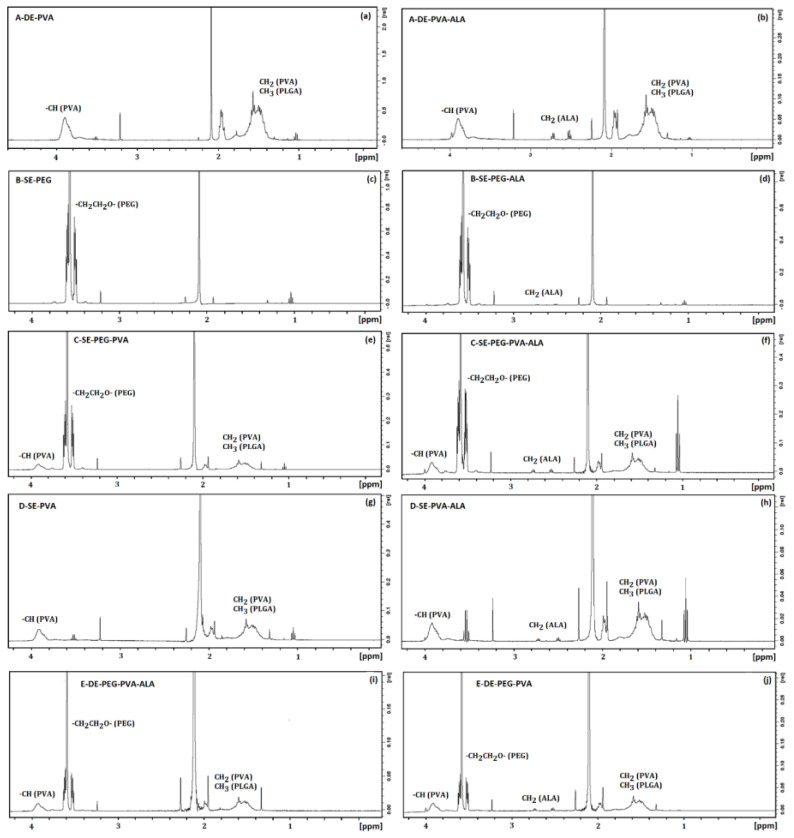
NMR spectra of (**a**) A–DE–PVA, (**b**) A–DE–PVA–ALA, (**c**) B–SE–PEG, (**d**) B–SE–PEG–ALA, (**e**) C–SE–PEG–PVA, (**f**) C–SE–PEG–PVA–ALA, (**g**) D–SE–PEG–PVA, (**h**) D–SE–PEG–PVA–ALA, (**i**) E–DE–PEG–PVA, (**j**) E–DE–PEG–PVA–ALA.

The spectra of System A–DE–PVA (Figure 6a) showed a peak from 3.9 ppm to 4.1 ppm assigned to the methine proton of PVA (–CH). The broad peak at 1.50 ppm represented the overlapped –CH_3_ and –CH_2_ groups from PLGA and PVA molecular structures, respectively [62,63,64,65]. Figure 6b shows the spectra of System A–DE–PVA–ALA, and the addition of 5-ALA in the PVA–PLGA carriers was confirmed by the peaks at 2.7 ppm and 2.9 ppm, which were related to the –CH_2_ group [66]. The proton FID from –CH_2_ (PVA) and –CH (PVA) groups was performed before and after the interaction with 5–ALA. The active induced a slight increase in the T1 values of PVA, reducing the rotation rate of the surfactant methylenes [67,68].

The spectrum of System B–SE–PEG (Figure 6c) shows a sharp peak at 3.65 ppm assigned to –CH_2_CH_2_O groups from PEG. This peak was also found in the spectrum of System B–SE–PEG–ALA (Figure 6d), also presenting typical peaks of 5-ALA at ~2.7 ppm (t) and –CH_2_ group at ~2.90 ppm (t). Considering the proton FID from the −CH_2_CH_2_O groups of PEG before and after addition of 5–ALA, the T1(s) times decreased by 8.42%. This result suggested that 5−ALA increased the rotational mobility in the PEG region.

The spectra of Systems C–SE–PEG–PVA and C–SE–PEG–PVA–ALA are shown in Figure 6e,f, respectively. The sharp peak at 3.65 ppm was assigned to the –CH_2_CH_2_O groups of PEG, and between 3.9 ppm and 4.1 ppm was related to the structure of PVA [63,64]. The broad peak at 1.50 ppm represented an overlapping of the –CH_3_– and CH_2_– groups from PLGA and PVA molecular structures, respectively [62,63,64,65]. The spectrum of System C–SE–PEG–PVA–ALA presented the typical proton peaks of –CH_2_ and CH_2_ groups of 5-ALA, respectively, at ~2.7 ppm (t) and ~2.90 ppm (t) [66].

The spectra of Systems D–SE–PEG–PVA and D–SE–PEG–PVA–ALA are shown in Figure 6g,h, respectively. The peak at 3.65 ppm was assigned to the –CH_2_CH_2_ groups. The peak of the methylene group of PEG between 3.9 ppm and 4.1 ppm was also found in the PVA structure. The broad peak at 1.50 ppm represented an overlapping of the –CH_3_ and –CH_2_ groups of the PLGA and PVA molecular structures, respectively. The spectrum of System D–SE–PEG–PVA–ALA also presented typical proton peaks of the –CH_2_ group from 5-ALA at ~2.7 ppm (t) and ~2.90 ppm (t), respectively.

The results from the simple emulsion systems indicated that 5-ALA slightly influenced all groups, reducing the T1 values of the PVA groups and marginally increasing that of the PEG group. Thus, 5-ALA disordered the PVA groups but presented a slightly restricted effect on the PEG groups, indicating a significant interaction between 5-ALA and PVA.

The 5-ALA reduced the T1 values of PVA and PLGA in the double emulsion systems due to the decrease in the molecular packing in the hydrophobic region. This effect was opposite to that related to System A–DE–PVA, probably due to the influence of PEG because similar results were observed by UV–VIS spectroscopy. Therefore, our results suggested competition between PVA and PEG carriers to interact with 5-ALA. The active interacted more efficiently with methylene regions in the presence of the PEG carrier due to the space between the formed surface layers, allowing a more efficient insertion of 5-ALA into the E–DE–PEG–PVA system. For this reason, the 5-ALA was more available when interacting with PEG, probably resulting in a slower release.

These interactions were better evaluated when the spectra of the double emulsion of Systems A and D were compared (Figure 6). The peak found in the range from 3.9 ppm to 4.1 ppm was attributed to the methine proton of PVA (–CH), and the broad peak at 1.50 ppm represented an overlapping of the –CH_3_ and –CH_2_ groups from PLGA and PVA structures, respectively. The spectrum of the double emulsion D–DE–PEG–PVA also showed a large peak at 3.65 ppm, which was assigned to the –CH_2_CH_2_O group from PEG. The insertion of PEG into the PVA–PLGA particles increased the T1 values of –CH_2_ and –CH groups from PVA around 32% and 3.7%, respectively. For this reason, PEG decreased the rotational movement of the PVA groups in the system.

#### 3.2.7. Forced Stability of 5-ALA

**Figure 7:**
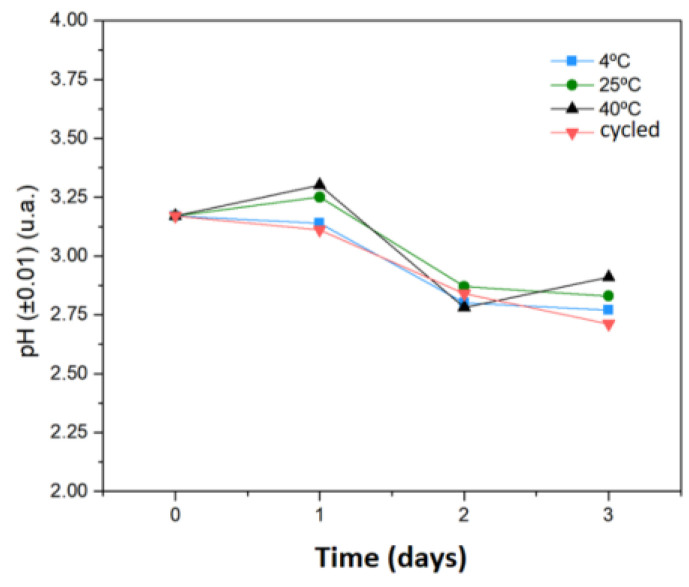
Forced stability of 5-ALA in aqueous medium for 4 days at 4 °C, 25 °C, 40 °C and cycled (24 h at 4 °C and 24 h at 40 °C) conditions.

Figure 7 shows that the evaluated concentration and temperature marginally influenced the pH values. Furthermore, the final evaluation showed no change in color. According to a previous report, a 5-ALA solution (0.3 g.mL^−1^; pH = 1.7) did not show significant changes in pH after 6 weeks at 40 °C, while a degradation of 5-ALA (5%) was observed in pure water (no pH adjustment) under the same storage conditions [69]. 

As reported elsewhere [70], the pH values is considerably acidic for most systems containing encapsulated 5-ALA, influencing the conversion/production of protoporphyrin IX. The pH of the medium can be increased from 7 to 7.5, allowing the enhanced production of protoporphyrin IX, but the physiological pH value can influence the systems’ stability since 5-ALA undergoes dimerization at this pH value. Moreover, this formulation showed greater stability (37 h) at pH = 3.0 (or lower) than those maintained at pH = 7 and 50 °C. However, these results are dependent on the concentration of 5-ALA in solution and on the storage temperature: the lower the concentration at physiological pH, the lower the degradation of 5-ALA [7,71].

According to a previous report [71], when 5-ALA was maintained at pH = 2.35, its amino group was protonated and did not react with the ketone group from other molecules in the medium, preventing dimerization. A higher concentration of protons in the medium (related to the reaction time) decreased the pH values and changed the color of the solution to amber-yellow. For this reason, the pH of the particle solutions was maintained at acidic pH to reduce the degradation process.

#### 3.2.8. Forced Stability of Nanoemulsions (3000 rpm and 56 °C)

Nanoemulsions presented pH stability after three centrifugation cycles at 3000 rpm for 30 min. System D (D–SE–PVA) and System E (E–DE–PEG–PVA) showed the greatest pH variation, of 3.72% and 8.03%, respectively. The loaded systems did not present precipitates and no significant pH variation in the presence of PEG–PVA. System B (B–SE–PEG) was the least unstable. The differences between the initial and final pH were 4.46%, 4.08% and 1.42%, respectively, for Systems B–SE–PEG–ALA, C–SE–PEG–PVA–ALA and E–DE–PEG–PVA–ALA. Only System D (D–SE–PVA) showed a considerable amount of precipitate, while System E (E–DE–PEG–PVA) did not show any precipitate despite presenting higher turbidity and phase separation. The centrifugation evaluation was not an exclusion because no destabilization was observed.

The systems were subjected to forced stability as a function of time and extreme temperature (56 °C) [19]. After 48 h, the emulsions presented sedimentation confirmed by the increase in the size distribution. Temperature changes influence pH and zeta potential (due to the presence of functional groups or ionic species in the medium), as well as the particle surface [72]. The final evaluation showed that the systems containing PVA presented higher translucence. Moreover, a precipitate was observed in the systems PVA–PEG. However, the forced stability evaluation as a function of time and extreme temperature was not considered here as an exclusion test.

The encapsulated 5-ALA was considered more stable when compared to the free one. This results show the efficiency of nanostructured systems in the protection of hydrophilic, active molecules. Similar results were observed in other reports on the encapsulation of 5-ALA and chitosan matrices [17].

#### 3.2.9. Influence of Storage Temperature on Physicochemical Stability

We evaluated the influence of the storage temperature (4 °C, 25 °C, 40°C and a cycled from 4 °C to 40 °C) on physicochemical stability after 40, 60 and 720 days. The loaded particles were influenced by the presence of 5-ALA, as expected. Besides presenting more acid systems, this result corroborates the electrical conductivity measurements, as well as the FTIR and ^1^H-NMR analyses.

All systems presented higher acidity during the analyses, regardless of the presence of 5-ALA. At 40 °C, the systems presented translucence and precipitates. This characteristic was better observed in Systems A, D and E. After 7 days, a difference in pH values was observed, which was related to particle agglomeration.

The systems containing 5-ALA were more stable over time at room temperature. After 40 days, Systems A and E presented phase separation. After magnetic stirring, these systems became monophasic again for 3 h. For this reason, this result was not considered as a destabilization. Similar behavior was found in the analyses at 4 °C: although a stable behavior was observed between 7 and 40 days, the decay became more unstable over 60 days, with greater variation in System C containing 5-ALA. Changes in pH from 360 and 720 days were observed in Systems A, C and D containing 5-ALA.

The cycled systems presented translucence in the final evaluation. Systems A and E, as well as System C containing 5-ALA, presented precipitates. System D was more stable than System A, while Systems C and E presented similar stability over time. Therefore, the type of emulsion (single or double) implied important changes.

In general, the particles presented reduced size over time, and the systems containing 5-ALA presented higher stability. Colloidal stability at 40 °C occurred from 7 to 30 days, when the size variation was not observed in System D–SE–PVA–ALA, and from 7 to 20 days in System C–SE–PEG–PVA–ALA. After 40 days, higher instability was noticed in System B–SE–PEG–ALA, showing that interactions allowing particle stabilization were not sufficient. For the cycle conditions, the systems presented behavior similar to that at 40 °C.

The systems presented more stability over time at 25 °C. System A containing 5-ALA remained constant, although it showed phase separation. System C did not show particle size variation. For Systems C and E, the synthesis method influenced the stability.

The size variation in the systems stored at 4 °C was similar to that at 25 °C, although the greatest variation was observed after 100 days, which was related to particle swelling.

All systems presented initial PDI values below 0.2. However, these values increased to 1.1 when they were stored at 40 °C. The systems stored at 25 °C did not show PDI changes over time, remaining below 0.4. However, the systems stored at 4 °C and cycled presented PDI values below 1.0. Lower PDI values (from 0.1 to 0.2) are related to systems presenting more homogeneous particle size distributions [73].

The PVA solution (25 °C) presented surface charge of (–0.07 ± 2.26) mV, while at 0 °C, a reduced value of (–9.82 ± 2.26) mV was observed. For this reason, the synthesis temperature influenced the systems’ stabilization by PVA. The surface charge was directly influenced by the addition of PEG when it was added to the PVA solution (0 °C; −0.63 ± 3.82 mV). However, previous analyses showed that 5-ALA influenced the systems’ stabilization as a result of the different surface charges of the unloaded and loaded systems.

Surface charge variation over time was observed at 40 °C due to the systems’ destabilization (phase separation, precipitate, reduced pH and particle swelling). Systems A, C and D presented charge variation similar to that of the initial values at 25 °C. System C–SE–PEG–PVA–ALA presented similar behavior at 4 °C when compared to that at room temperature. The charge stabilization after the cycle evaluation presented similar behavior when compared to those at 40 °C and 4 °C.

A previous report showed that the physicochemical properties of PLGA did not change at temperatures considered herein [74]. However, storage at 40 °C favored faster evaporation of the residual solvent. Moreover, the PLGA mass (50:50) did not favor aggregate formation. Previous work presented similar results at 4 °C and 40 °C considering the same polymer particles containing paclitaxel (non-ionic and hydrophobic), and particle aggregation was observed at the highest temperature.

PLGA copolymers may suffer surface erosion under some conditions, allowing degradation and modification of the physicochemical parameters [75]. On the other hand, PEG is a source of protons [76], so the system stabilization can be related to the polymer concentration. The observed changes suggested a dissociation of carboxylic groups from the water/particle interface due to relaxation of the polymeric chains. In this case, the particles’ hydrodynamic diameter was increased in the presence of active, leading to its exposure to the medium, modifying the surface charge, pH and PDI values [72].

The emulsions kept the 5-ALA more stable over time when compared to its unloaded form, resulting in promising delivery systems for this drug. For this reason, degradation over time can be avoided, as suggested elsewhere [17].

#### 3.2.10. IDMAP Tool Analysis

The results related to (i) the global analysis of the systems at 4 °C, (ii) the data analysis of unloaded and loaded particles, and (iii) the influence of surfactants on each component of the formulation were mapped by the ideal mapping technique (IDMAP) [77].

**Figure 8:**
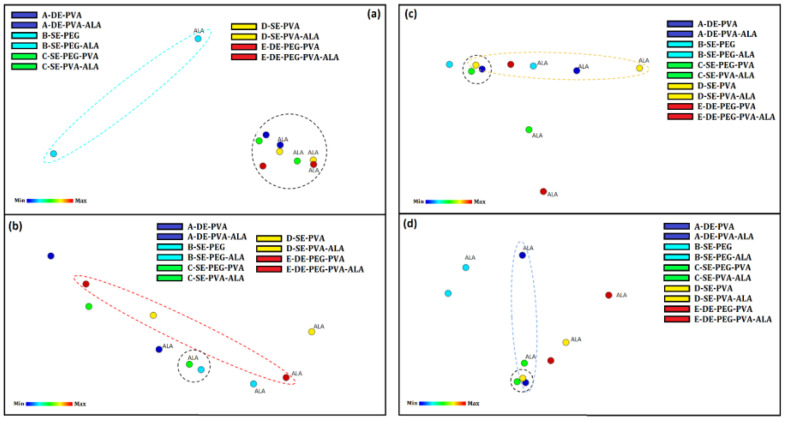
(**a**) IDMAP maps for zeta potential analysis; (**b**) IDMAP maps for pH analysis; (**c**) IDMAP maps for the particle size and (**d**) IDMAP maps for the PDI of System A (A–DE–PVA and A–DE–PVA–ALA), System B (B–SE–PEG and B–SE–PEG–ALA), System C (C–SE–PEG–PVA and C–SE–PEG–PVA–ALA), System D (D–SE–PVA and D–SE–PVA–ALA) and System E (E–DE–PEG–PVA and E–DE–PEG–PVA–ALA) showing the similarities of the proximity systems.

Figure 8 shows the IDMAP maps considering the zeta potential analysis of the systems stabilized with PVA. The 5-ALA marginally influenced this group, as expected. Thus, the systems stabilized with PVA, as well as the combination PEG–PVA, were similar in terms of stability. PVA was more influenced when compared to PEG400 for loaded particles. Furthermore, PEG400 showed great differentiation due to the influence of the active, which was confirmed by the separation of the B–SE–PEG group.

The pH evaluation (Figure 8b) revealed that both unloaded and loaded systems containing PVA–PEG were vertically differentiated. System B–SE–PEG was marginally influenced by the presence of 5-ALA, while the opposite relationship was observed in System E–DE–PEG–PVA. The other systems were similarly influenced by 5-ALA. The increase in acidity indicated stability. In addition, the 5-ALA kept the pH slightly more stable over time, while the unloaded systems presented significant variation (decreased pH) during the evaluation time.

The particle size evaluation (Figure 8c) showed that 5-ALA influenced the increased hydrodynamic diameter, suggesting that the active may be located inside or adsorbed by the particles, explaining the increased volume. However, the hypothesis supported by the surface analysis was that 5-ALA was adsorbed in the particle matrix.

The systems containing PEG and PEG–PVA were less influenced by the presence of 5-ALA. Moreover, PEG and PVA influenced, respectively, the unloaded and loaded particle sizes due to the greater interaction of 5-ALA and PEG chains (as suggested by the surface analysis).

Systems A, C, D and E presented similar particle diameters, and the influence from 5-ALA was similar in all systems and more pronounced in System D–SE–PVA. For temporal analyses, the decrease in the particle size over time may indicate exposure of the medium to 5-ALA. The most stable systems were C–SE–PVA–PEG and E–DE–PVA–PEG.

The PDI evaluation (Figure 8d) showed no significant differences among unloaded particles. However, PEG marginally influenced the unloaded and loaded particles. The loaded particles stabilized by PVA presented more differentiation. The simple emulsion systems presented less difference in relation to the presence of 5-ALA. When globally compared, Systems D–SE–PVA and E–DE–PVA–PEG presented better stability. When compared to Systems A–DE–PVA and B–SE–PEG, Systems D–SE–PVA and C–SE–PEG–PVA were marginally influenced by 5-ALA. For this reason, System C–SE–PVA–PEG presented greater stability and less influence from 5-ALA. Accordingly, this system was identified as a prominent candidate for successful encapsulation of 5-ALA that may allow improved skin permeation.

## 4. Conclusions

Five different systems were developed based on the type of stabilization (PVA, PEG or PEG–PVA) and method of production (single or double emulsion). Surface analysis allowed us to verify that 5-ALA was adsorbed on the particle surfaces, in addition to interacting with PEG and PVA. The 5-ALA presented considerable affinity of interaction in the particles stabilized with PEG. The temporal stability evaluation showed that the systems stabilized with the combination PEG–PVA presented good stability at 4 °C and 23 °C due to their lower variation in size and the results of surface charge analyses. Another contribution of this work was the proposal of a stable system representing a promising new pharmaceutical product. The IDMAP tool allowed a more comprehensive data analysis, indicating that a better developed system had been obtained by simple emulsion and stabilized with the combination PEG–PVA, as confirmed by all reported previous results.

## Figures and Tables

**Table 1:** Surface tension (*γ*) of probe liquids for surface energy calculation.

Probe Liquid	*γ_total_*	*γ_dispersive_*	*γ_polar_*	*γ_acid_*	*γ_basic_*
water	72.80	21.08	51.72	25.50	25.50
formamide	58.00	39.00	19.00	2.28	39.60
ethylene glycol	48.00	29.00	19.00	3.00	30.10
diiodomethane	50.80	48.50	2.300	0.00	0.00

**Table 2:** Systems development.

Emulsion Method	Sample	Internal Aqueous Phase	External Aqueous Phase	Observation
(A) Double	A–DE–PVA	water	PVA	***Literature and method review*** Control of Synthesis E, Comparison to System D
	A–DE–PVA–ALA	5-ALA	PVA	
(B) Simple	B–SE–PEG	-	PEG	***Method review*** Control of Synthesis C and E
	B–SE–PEG–ALA	-	PEG–ALA	
(C) Simple	C–SE–PEG–PVA	-	PVA–PEG	***Innovation*** Comparison to System E
	C–SE–PEG–PVA–ALA	-	PVA–PEG–ALA	
(D) Simple	D–SE–PVA	-	PVA	***Method review*** Modified synthesis from (A) Comparison to System A System E control.
	D–SE–PVA–ALA	-	PVA–ALA	
(E) Double	E–DE–PEG–PVA	PVA	PEG	***Innovation*** Comparison to System C.
	E–DE-PEG–PVA–ALA	PVA–ALA	PEG	

**Table 3:** Hydrodynamic size comparison of System A (A–DE–PVA and A–DE–PVA–ALA), System C (C–SE–PEG–PVA and C–SE–PEG–PVA–ALA), system D (D–SE–PVA and D–SE–PVA–ALA) and System E (E–DE–PEG–PVA and E–DE–PEG–PVA–ALA) obtained by NTA and DLS techniques.

System	Average Size NTA (nm)	Mode (nm)	Standard Error (nm)	Average Size (DLS) (nm)	Particle mL^−1^
A–DE–PVA	(225.7 ± 0.7)	(218.3 ± 12.8)	(33.5 ± 1.2)	(226.4 ± 70.2)	1.99 × 10^12^ ± 3.74 × 10^10^
A–DE–PVA–ALA	(192.7 ± 6,5)	(195.1 ± 7.9)	(24.7 ± 4.7)	(195.9 ± 61.2)	1.79 × 10^11^ ± 1.27 × 10^10^
C–SE–PEG–PVA	(153.9 ± 1.3)	(149.0 ± 2.7)	(24.8 ± 3.4)	(151.6 ± 49.0)	1.53 × 10^14^ ± 5.62 × 10^12^
C–SE–PEG–PVA–ALA	(334.4 ± 4.0)	(316.3 ± 8.8)	(70.4 ± 3.8)	(377.4 ± 62.6)	1.85 × 10^13^ ± 7.92 × 10^11^
D–SE–PVA	(142.6 ± 0.9)	(137.9 ± 1.9)	(22.5 ± 1.3)	(181.1 ± 51.2)	2.36 × 10^15^ ± 5.69 × 10^13^
D–SE–PVA–ALA	(445.7 ± 18.5)	(341.9 ± 28.5)	(149.9 ± 7.4)	(702.1 ± 118.9)	7.97 × 10^11^ ± 6.10 × 10^10^
E–DE–PEG–PVA	(173.2 ± 0.8)	(165.3 ± 3.5)	(44.2 ± 0.6)	(199.4 ± 60.8)	3.50 × 10^14^ ± 7.4 × 10^12^
E–DE–PEG–PVA–ALA	(164.5 ± 4.2)	(153.8 ± 1.9)	(23.3 ± 3.3)	(274.6 ± 117.5)	9.23 × 10^11^ ± 6.12 × 10^10^

## Data Availability

Not applicable.
